# Validation of the attitudes to patient safety questionnaire for nursing students in the Spanish context

**DOI:** 10.1186/s12912-021-00634-y

**Published:** 2021-06-19

**Authors:** Águeda Cervera-Gasch, Víctor M González-Chordá, Fred Gustavo Manrique-Abril, Laura Andreu-Pejo, María Jesús Valero-Chillerón, Desirée Mena-Tudela

**Affiliations:** 1 Nursing Department, Univesitat Jaume I, Avda Sos Baynat s/n., 12071 Castellón, Spain; 2 Nursing Department, Universidad Nacional de Colombia, Carretera 45, Bogotá, Colombia

**Keywords:** Nursing education, Patient safety, Validation Studies, Attitudes

## Abstract

**Background:**

There are different instruments to assess the attitudes of nursing students towards patient safety. However, no questionnaire validated in Spanish with this objective was identified. The objective of this study was to validate the Attitudes to Patient Safety (APS) questionnaire for nursing students in Spain and to study the attitudes towards patient safety of nursing students at the Universitat Jaume I (Spain).

**Design:**

Cross-sectional psychometric study developed in a sample of 177 undergraduate nursing students. The study was carried out in the second semester of 2016.

**Methods:**

First, a nominal group was created to perform cross-cultural adaptation and determine content validity of the Attitude to Patient Safety Questionnaire (APQS-III). Second, a cross-sectional study was conducted to determine the psychometric properties of the questionnaire and to study nursing student attitudes towards patient safety.

**Results:**

Exploratory factorial analysis explained 53.82 % of the variance, with good internal consistency (α = 0.808), and confirmatory factor analysis indicate an adequate fit between the model and the data (χ2 = 366; *p* < 0.001; χ2/df = 1.886; RMSEA = 0.07; IC95 %=0.059–0.081; CFI = 0.885). Intra-observer reliability was good (ICC = 0.792, *p* < 0.001). The mean overall score of the questionnaire was 3.92 (95 % CI = 3.88–4.03). Significant differences were observed regarding whether the students had completed a clinical practicum (*p* = 0.012) and the academic year (*p* = 0.25).

**Conclusions:**

The psychometric properties of the APS questionnaire adapted for Spanish nursing students are adequate. Students show an adequate attitude towards patient safety; however, it is necessary to develop a strategy to guarantee the acquisition of competency for patient safety as well as to design and evaluate specific educational interventions.

**Supplementary Information:**

The online version contains supplementary material available at 10.1186/s12912-021-00634-y.

## Background

The education of future health professionals, specifically nursing professionals, is considered a key element to address the challenge of patient safety. In fact, the World Health Organization published recommendations in 2011 with 11 curricular topics to guide education on patient safety [1]. In the USA, the Quality and Safety Education for Nurses (QSEN) initiative was developed, establishing a framework with 6 competencies that should be included in nursing curricula [2]. In Europe, there is no similar strategy available to improve patient safety education for future nurses [3]. In Spain, nursing education is regulated by ORDER CIN / 2134/2008, of July 3, on the requirements for verification of official university degrees that qualify for the practice of the nursing profession [4]. This Order establishes the duration of the study plans (four years and 240 European Credit Transfer System -ECTS-) and the competences to be acquired. Specifically, three of these competences address the term safety in a generic way, although none of them makes express mention of patient safety. However, each university is free to develop its own curriculum and complement these competences with others or develop them as learning outcomes. In fact, Mira et al. [5] concluded that, in Spain, it is necessary to review the curricula, the objectives of clinical practicums and teaching methodologies to address competencies in patient safety.

The available evidence does not clarify the most appropriate strategies for incorporating patient safety into nursing curricula [3, 6] or the most effective educational interventions to ensure the acquisition of competencies [7, 8]. Similarly, there is a gap in the evaluation of knowledge, skills and attitudes about patient safety [9], making it necessary to have validated tools to determine which are the most appropriate curricular strategies and educational interventions.

Okuyama et al. [9] conducted a systematic review to identify the tools available for evaluating competencies on patient safety in health science professionals and students. The authors identified a total of 34 tools and concluded that none of them covered all the competencies related to patient safety and competency levels according to Miller’s pyramid, making it necessary to combine different tools to obtain a reliable and complete evaluation. Specifically, the authors only found 2 instruments [10, 11] with adequate validity and reliability to evaluate knowledge on patient safety in nursing students.

In 2010, Chenot et al. [12] developed and validated the Health Care Professionals Patient Safety Assessment Curriculum Survey (HPPSACS) in the United States based on the competency framework of the QSEN strategy [2]. In 2015, Mansour [13] adapted this questionnaire for nursing students in the United Kingdom. Additionally, Ginsburg et al. [14] validated the Health Professional Education in Patient Safety Survey (H-PEPSS) to evaluate competencies in patient safety in a Canadian sample of 1,247 recent graduates in pharmacy, medicine and nursing. Subsequently, the H-PEPSS questionnaire was modified by Luckewich et al. [15] and adapted by Bressan et al. [16] for Italian nursing students. In Korea, Lee et al. [17] developed and validated the Patient Safety Competency Self-evaluation (PSCSE) questionnaire using a sample of 354 nursing students. Tella et al. [18] created the Patient Safety in Nursing Education Questionnaire (PaSNEQ) to compare the perception of English and Finnish nursing students on the acquisition of skills related to patient safety during clinical practicums. Other authors validated more specific instruments to study the notification of errors by nursing students [19] or to evaluate specific educational interventions on patient safety [20–22].

In recent years, the number of publications on this topic has increased, and it is possible to find new validated instruments to evaluate the knowledge, skills and attitudes about patient safety in nursing students. In general, the psychometric properties of these instruments were good when they were reported and validated in English or Asian countries. At the time of the study, no questionnaire validated in Spanish was identified for use in nursing students.

However, there are questionnaires used in students of other health sciences that have been validated in Spanish. Specifically, the original version of the Attitudes to Patient Safety (APS) questionnaire [23] was adapted for Latin American medical students by Lamponi et al. [24], with Cronbach alpha values ranging from 0.76 to 0.88. This questionnaire was originally developed and validated by Carruters et al. [23] in the United Kingdom to study attitudes towards patient safety in medical students. The APS questionnaire is composed of 26 items organized into 9 dimensions (Patient safety training received, Error reporting confidence, Working hours as the cause of errors, Error inevitability, Professional incompetence as the cause of errors, Disclosure responsibility, Team functioning, Patient involvement in reducing errors, and Importance of patient safety in the curriculum). The original version of the APS questionnaire revealed good stability of its factorial structure (reliability coefficients of the dimensions between 0.64 and 0.82) and adequate content validity (α = 0.73). The authors of the questionnaire concluded that this tool can be used to measure attitudes towards patient safety in health science students in other contexts, in addition to evaluating changes in the curriculum.

In fact, the APS questionnaire has been used in different studies to measure the attitudes of medical students in the United States [25], Germany [26] and Pakistan [27], among other countries, with adequate psychometric properties whenever they were reported. Raines et al. [28] used the APS questionnaire to evaluate the effectiveness of an educational intervention in a sample of 60 master students in nursing. The authors state that they used a version adapted for different health professions; however, they do not show the items, the psychometric properties or the validation process.

After reviewing that the APS questionnaire has been widely used with medical students and has adequate psychometric properties, it was decided to validate the Latin American version of this questionnaire [24] for nursing students in Spain and to study attitudes of nursing degree students towards patient safety at Universitat Jaume I (Spain).

## Methods

### Design and setting

A validation study of the APS questionnaire for nursing students in the Spanish context was conducted. First, a nominal group was created to perform cross-cultural adaptation and determine content validity. Second, a cross-sectional study was conducted among nursing students at Universitat Jaume I (Spain) to determine the psychometric properties of the questionnaire and to study the students’ attitudes towards patient safety. The study was conducted in the second semester of 2016.

The nursing degree at Universitat Jaume I (UJI) (Spain) started in 2011, once the European Higher Education Area (EHEA) was established [29]. The program has four courses and includes the minimum competencies established in Spanish legislation [4]. Contents related to patient safety are specifically addressed in the third year, within the subject “Management of care in the socio-sanitary field”, with a total of six ECTS.

### Transcultural adaptation and content validity

A nominal group consisting of 4 nursing professors with previous experience in patient safety, care quality and instrument validation and 2 nursing students in their final year reviewed the Latin American version of the APS questionnaire [24] to identify semantic differences and transculturally adapt the questionnaire to the Spanish context and to the field of nursing.

The members of the nominal group received an email with an invitation and prior information on the methodology and objectives of the study, the Latin American version of the APS questionnaire [24] and an informed consent form. Information was collected through a face-to-face meeting that was voice recorded. During the meeting, the wording of each dimension and item and its suitability to the Spanish context and to the field of nursing were analysed, reaching a consensus on its content validity. Participants also had the opportunity to propose new items. The final questionnaire was written and distributed to the members of the nominal group by email requesting a new evaluation. No additional observations were made.

### Psychometric properties and attitudes of nursing students regarding patient safety

A cross-sectional study was conducted. The study population consisted of 240 nursing students at Universitat Jaume I (Spain) (60 students per course). The students enrolled in any of the four nursing degree courses who were present on the day of data collection and voluntarily wanted to complete the questionnaire were included. Questionnaires that were not fully filled were excluded. Convenience sampling was conducted and a sample size between 5 and 10 subjects per item was considered sufficient [30].

The version of the APS questionnaire adapted by the nominal group was administered during normal classes. Questions to obtain sociodemographic data (age and gender), academic year, previous studies related to health sciences (yes, no) and clinical practicums were included. The students received prior information on the objectives and methodology of the study and were informed of its voluntary and anonymous nature.

As in previous studies [31, 32] and because we started from a questionnaire already validated, construct validity was analysed with exploratory and confirmatory factorial analysis. Firstly, the original structure of the APS questionnaire was tried to replicate with an exploratory factor analysis (EFA) using the principal component method with varimax rotation. A minimum factor load of 0.3 was established as a threshold to retain an item within a certain factor [33]. Internal consistency was measured using Cronbach’s alpha (α) and the possibility of removing an item was assessed if the item-total correlation was less than 0.4 and increased in α value in the corresponding dimension [34]. However, as shown in the Results section, the EFA showed a new factorial structure composed of 6 dimensions compared to the original 9-dimensional structure.

Secondly, a confirmatory factorial analysis (CFA) was performed with the same sample to test a six-factor model using the maximum likelihood estimation technique. According with Kline [35], the goodness of fit was studied by means of the chi-square (χ2, small scores indicate good fit), ratio of χ2 to degrees of freedom (χ2/df < 0.5 indicates an adequate fit), Root Mean Square Error of Approximation (RMSEA ≤ 0.1 indicates an adequate fit) and Comparative Fit Index (CFI ≥ 0.90 indicates a good fit). Intraobserver reliability was determined with the intraclass correlation coefficient (ICC) in a sample of 20 students (2 measurements were performed with a separation of 2 weeks).

In addition, a descriptive analysis of the questionnaire was performed using means and standard deviations. Likert scale scores were reversed for items written negatively. The item scores were added and divided by the number of items to obtain the mean score for each dimension and the overall score [36]. The relationships between the variables studied and the total score of the questionnaire, its dimensions and items were studied. Normality was analysed with the Kolmogorov-Smirnov test, and to determine statistical significance, the non-parametric Mann-Whitney U test (2 groups) and the Kruskal-Wallis test (3 or more groups) were used. A *p*-value < 0.05 was considered statistically significant in the hypothesis testing. The statistical analysis was carried out with SPSS v23.

### Ethical considerations

This study was authorized by the Nursing Department of the Universitat Jaume I. The questionnaires did not include personal data that would allow the identification of the participants. The students received prior information about the voluntary and anonymous nature of the study and all of them gave their informed consent to participate in the study. The experts participated voluntarily in the study with prior informed consent. Permission from the authors of the original APS questionnaire [23] and from the authors of the adapted version in the Latin American context [24] was requested by email. The project was designed in accordance with the Organic Law 15/1999 of 13 December on Protection of Personal Data, and the ethical principles of the Declaration of Helsinki (beneficence, nonmaleficence, autonomy and justice) were respected.

## Results

### Transcultural adaptation and content validity

The nominal group made minor changes in expressions to favour semantic equivalence, and the wording was adapted to the field of nursing studies (for example, the term doctor was changed to nurse). There was also consensus on changes in the structure of the questionnaire. In this way, a questionnaire composed of 29 items (26 items in the original questionnaire) was obtained, organized into 9 dimensions. Additional file 1 offers a comparison of the items and dimensions of the original APS questionnaire [23], the adapted version for medical students in Spanish [24] and the version adapted for nursing students by the nominal group. It was agreed to use a 5-point Likert scale (1 = strongly disagree; 5 = strongly agree) to answer the items.

### Psychometric properties

The APS questionnaire (version adapted by the nominal group) was administered to a sample of 177 nursing students to determine its psychometric properties and the attitude of the students towards patient safety. No questionnaires were excluded for not being fully filled. The mean age of the sample was 22.59 (± 5.915) years, 77.4 % (*n* = 137) were women, and 78 % (*n* = 138) had no previous studies related to the health field. A total of 79.7 % (*n* = 141) had completed a clinical practicum (Table 1).

**Table 1:** Sample description

Variables	Sample	Courses			
		**First**	**Second**	**Third**	**Fourth**
Age (m; SD)	22.6(5.915)	22.17(8.8)	23.18(6.44)	22(3.91)	22.78(2.16)
Course (n; %)	177(100)	23.2(41)	55(31.1)	40(22.6)	41(23.2)
Gender (n; %) Man Woman	40(22.6) 137(77.4)	28(20.4) 13(32.5)	7(17.5) 48(35)	11(27.5) 29(21.2)	9(22.5) 32(23.4)
Previous studies (n; %) No Yes	138(77.9) 39(22.1)	32(23.2) 9(23.1)	42(30.4) 13(33.3)	32(23.2) 8(20.5)	32(23.2) 9(23.1)
Clinical practicum (n; %) No Yes	36(20.3) 141(79.7)	34(94.4) 7(5)	2(5.6) 53(37.6)	0 40(28.4)	0 41(29)

The Kaiser-Meyer-Olkin (KMO) test (KMO = 0.717) and Bartlett’s test of sphericity (*p* < 0.001) confirmed the applicability of the factor analysis. As a result, the questionnaire underwent modifications in its structure. The items were grouped into 6 dimensions that explained 53.82 % of the total cumulative variance. Some items changed dimensions; 5 items were eliminated to increase the internal consistency, and 2 were eliminated for having factorial loads lower than 0.3 and not being able to be grouped in any dimension. The overall internal consistency of the questionnaire with this new structure was α = 0.808 and showed good intraobserver reliability (ICC = 0.792; *p* < 0.001) (Table 2).

**Table 2:** Exploratory factor analysis and internal consistency results

Dimensions and items^a^	1	2	3	4	5	6	λ^b^	Α^c^
**Responsibility**							**15.29**	.**914**
Item 1	.**918**	− .016	.063	.066	.002	.018		.827
Item 2	.**948**	− .015	.011	.060	.016	− .005		.818
Item 3	.**949**	.041	.008	.000	− .023	− .016		.814
Item 4	.**935**	− .028	− .029	.028	.075	− .012		.820
Item 5	.**399**	.190	.386	− .252	.054	− .142		.896
**Organization and communication**							**14.42**	.**744**
Item 6	.079	.**775**	.064	.072	− .013	− .112		.651
Item 7	− .026	.**823**	.140	.022	.054	− .050		.630
Item 8	− .193	.**545**	.220	.066	.004	.321		.705
Item 9	− .105	.**552**	.256	.119	.035	.291		.687
Item 10	.087	.**471**	.357	− .126	− .020	− .240		.699
**Working as a team**							**7.13**	.**723**
Item 11	− .010	.020	.**769**	.057	.085	− .056		.638
Item 12	.037	.280	.**730**	.026	.106	− .013		.579
Item 13	− .063	.146	.**660**	.106	.055	− .202		.621
**Training**							**6.35**	.**535**
Item 14	− .054	.109	.385	.**463**	− .068	.158		.356
Item 15	− .095	.294	− .227	.**362**	.118	.028		.473
Item 16	.065	.243	.395	.**454**	.065	.077		.338
Item 17	− .025	.115	.112	.**709**	.089	− .122		.358
**Notification**							**5.78**	.**782**
Item 18	.033	− .010	.069	.112	.**860**	− .037		.380
Item 19	.124	− .091	.074	.049	.**818**	.004		.529
**Consciousness**							**4.83**	.**566**
Item 20	.292	.077	− .027	− .505	− .023	.**405**		.506
Item 21	.087	− .098	.031	− .032	− .126	.**754**		.360
Item 22	.063	.186	− .257	.014	.161	.**584**		.513

^a^: The statements of the dimensions and items are not included due to lack of space. They can be consulted in Table 3.

^b^: Percentage of variance explained by each dimension.

^c^: Cronbach’s alpha for each dimension and alpha if each item is eliminated

**Fig. 1:**
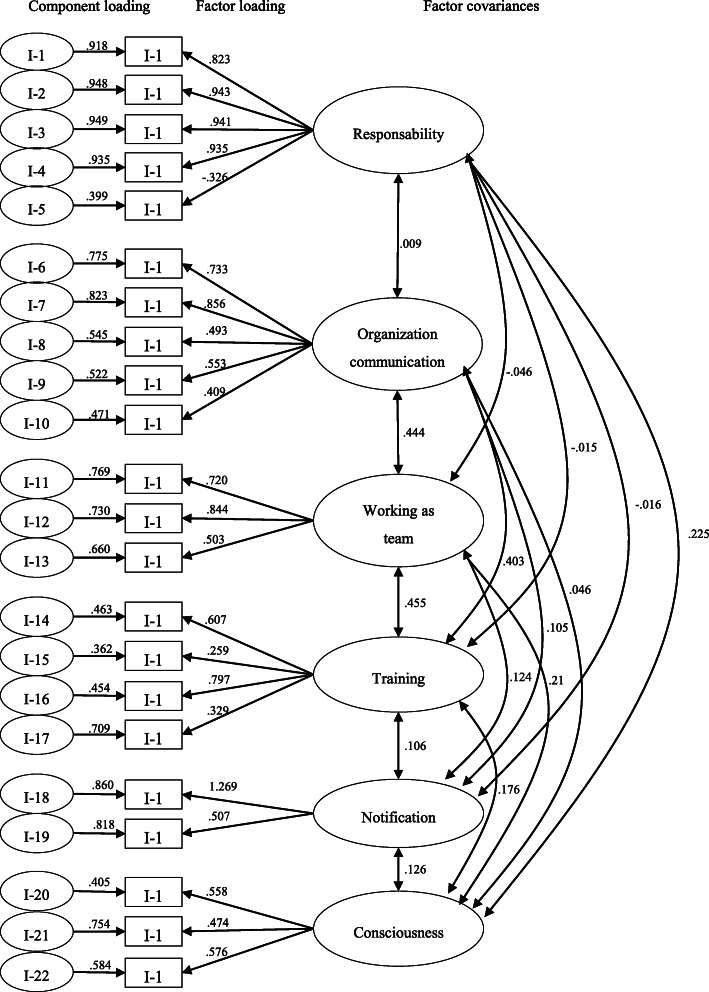
Path diagram

Figure 1 shows the path diagram after the confirmatory factor analysis. The result of χ2 (366; df = 194; *p* < 0.001), ratio of χ2 to degrees of freedom (χ2/df = 1.886), RMSEA (0.07, IC95 %=0.059–0.081) and CFI (0.885) indicate an adequate fit between the model structure and data.

### Attitudes of nursing students regarding patient safety

The mean overall score was 3.92 (95 % CI = 3.88–4.03) points. Table 3 shows the descriptive results by questionnaire dimension and item. No significant differences were identified in the overall score as a function of gender (U = 2471.5; *p* = 0.65) nor as a function of whether students had previous studies related to health sciences (X^2^ = 3.941; *p* = 0.715). On the other hand, students who had completed a clinical practicum obtained significantly higher overall scores (Me = 3.954; IQR = 0.36) compared to those who had not (Me = 3.818, IQR = 0.34) (U = 1853; *p* = 0.012). In addition, significant differences were observed for the overall score of the questionnaire as a function of the academic year (X^2^ = 9.323; *p* = 0.25), with second-year students obtaining higher scores (Me = 3.954; IQR = 0.32) (Table 4).

**Table 3:** Descriptive results of the APS questionnaire

Dimensions and items	m	95 % CI
**1. Responsibility**	3.62	3.53–3.70
Most errors derive from careless physicians^a^	3.55	3.43–3.67
Most errors derive from careless residents^a^	3.58	3.46–3.70
Most errors derive from careless nurses^a^	3.60	3.48–3.72
Most errors derive from careless nursing assistants^a^	3.59	3.47–3.70
If more attention was paid at work, errors would be avoided	3.80	3.7–3.89
**2. Organization and communication**	4.19	4.12–4.26
Adequate communication with the patient decreases adverse effects	4.03	3.92–4.13
Adequate communication with the team decreases adverse effects	4.24	4.15–4.34
An adequate workload decreases adverse effects	4.33	4.23–4.44
Adequate organization decreases adverse effects	4.40	4.30–4.49
The participation of patients in their care decreases adverse effects	3.99	3.90–4.08
**3. Teamwork**	4.26	4.19–4.33
Better work in a multidisciplinary team will reduce errors	4.26	4.17–4.35
Teaching teamwork skills will reduce errors	4.21	4.12–4.30
Learning about safety will allow me to become a more effective nurse	4.33	4.24–4.42
**4. Training**	4.01	3.95–4.08
My training prepares me to understand the causes of errors	4.00	3.91–4.09
I have a good understanding of patient safety issues due to my training	3.36	3.23–3.50
My training is preparing me to prevent errors in practice	4.09	4.00-4.19
The most experienced and competent nurses make mistakes	4.62	4.54–4.71
**5. Notification**	3.31	3.17–3.43
I would feel comfortable reporting any mistake I made	3.42	3.27–3.57
I would feel comfortable reporting other people’s mistakes	3.20	3.06–3.33
**6.Conciousness**	3.93	3.83–4.03
A true professional does not make mistakes ^a^	4.46	4.35–4.58
It is not necessary to inform the patient of errors that do not result in adverse effects^a^	3.79	3.64–3.93
Errors should be communicated to the patient only if they caused harm^a^	3.55	3.40–3.70
**Total APS**	**3.92**	**3.88–4.03**

^a^Items written in the opposite direction and with the scores inverted

In the analysis by dimension, the gender and previous studies variables showed no significant differences in any dimension (*p* > 0.05). Students who had completed a clinical practicum showed significantly higher scores in the dimensions Responsibility (U = 1973; *p* = 0.035) and Training (U = 1934; *p* = 0.025). Similarly, there were significant differences in the dimension Training (X^2^ = 10.746; *p* = 0.013) as a function of academic year, with an increasing score throughout the 4 academic years (Table 4).

In the analysis by item, some statistically significant differences were also found. The students who completed clinical practicums obtained higher mean scores for the items “Most errors derive from careless physicians” (Me = 3; IQR = 1; U = 1954; *p* = 0.022) and “Most errors derive from careless nursing assistants” (Me = 3; IQR = 1; U = 1999; *p* = 0.34) (Responsibility dimension) and for the items “An adequate workload decreases adverse effects” (Me = 4, IQR = 1; U = 1819; *p* = 0.004) (Organization and communication dimension) and “I have a good understanding of the safety issues of patients due to my training” (Me = 4; IQR = 1; U = 1475; *p* < 0.001) (Training dimension).

Finally, students with previous training in the field of health scored higher on the item “I have a good understanding of patient safety issues due to my training” (Me = 4; IQR = 1; X^2^ = 6.828; *p* = 0.33) (Training dimension), while students without prior training scored higher on the item “Errors should be communicated to the patient only if it caused harm” (Consciousness dimension) (Me = 4; IQR = 1; X^2^ = 6.047; *p* = 0.049). The score for the item “I have a good understanding of patient safety issues due to my training” increased significantly over the course of the 4 years, with fourth-year students obtaining the highest scores (Me = 4; IQR = 1; X^2^ = 18.795; *p* < 0.001).

**Table 4:** Results of the analysis of the questionnaire and its dimensions according to the variables studied

	Responsibility		Organization and communication		Teamwork		Training		Notification		Consciousness		Total	
	**Me**^**a**^	**p**	**Me**^**a**^	**p**	**Me**^**a**^	**p**	**Me**^**a**^	**p**	**Me**^**a**^	**p**	**Me**^**a**^	**p**	**Me**^**a**^	**p**
**Gender**		.076		.702		.388		.498		.124		.370		.345
Man Woman	3.2(.80) 3.8(.80)		4.2(.60) 4.2(.60)		4.1(3) 4.3(.67)		4(.50) 4(.50)		3.5(1) 3.5(1)		4(1) 4(.83)		3.8(.38) 3.9(.36)	
**Clinical practicum**		.035		.052		.469		.025		.835		.794		.012
No Yes	3.2(.95) 3.8(.80)		4(.80) 4.2(.60)		4.3(.67) 4.3(.67)		4(.75) 4(.50)		3.5(1.3) 3.5(1)		4(1.3) 4(.83)		3.8(.34) 3.9(.36)	
**Previous training**		.268		.808		.275		.736		.815		.049		.715
No Degree PT^b^	3.5(.80) 4.2(0) 3.8(2)		4(.60) 4.4(0) 4.2(.40)		4.3(.67) 4.1(0) 4(.67)		4(.50) 3.8(0) 4(.50)		3.5(1) 2.7(0) 3.2(1.5)		4(.67) 4.5(0) 3.6(.92)		3.9(.36) 4(0) 3.8(.40)	
**Academic year**		.156		.097		.592		.013		.500		.761		.025
First Second Third Fourth	3.2(.90) 3.8(.80) 3.7(.95) 3.8(.80)		4(.60) 4.2(.60) 4.2(.75) 4.2(.50)		4.3(.33) 4.3(.67) 4.3(.58) 4(.50)		4(.63) 4(.50) 4(.69) 4.2(.52)		3.5(1.7) 3.5(1) 3.5(3) 3.5(1.5)		4(1.1) 4(2.6) 4(.67) 4(.67)		3.8(.32) 3.9(.32) 3.9(.53) 3.9(.32)	

^a^ Results are expressed as the median and interquartile range (Q3-Q1), as determined by a non-parametric test

^b^ Professional training

## Discussion

The APS questionnaire for nursing students has adequate psychometric properties, with good construct validity, internal consistency, and temporal stability (Additional file 2). However, some dimensions of the questionnaire showed low internal consistency. These results coincide partially with those of Mansour [13], who studied the construct validity and internal consistency of the HPPSACS questionnaire, obtaining dimensions with Cronbach’s alpha values below 0.7. Factors such as systematic error [37] or the presence of atypical cases [38] can affect internal consistency, and Cronbach’s alpha values below 0.7 can be considered adequate [39]. It is possible that a transcultural adaptation, a more rigorous content validity analysis and a larger sample would have improved these results.

In addition, the questionnaire underwent significant modifications with respect to the original version [23] and the adapted version by Lamponi et al. [24]. In fact, Lamponi et al. [24] concluded that it is necessary to conduct studies with broader samples to confirm the factorial structure of the APS questionnaire. Thus, the original version of the APS questionnaire for medical students has 26 items organized in nine dimensions (Patient safety training received, Error reporting confidence, Working hours as the cause of errors, Error inevitability, Professional incompetence as the cause of errors, Disclosure responsibility, Team functioning, Patient involvement in reducing errors, and Importance of patient safety in the curriculum), while the version validated in this study for nursing students has 22 items organized in six dimensions (Responsibility, Organization and communication; Teamwork; Training; Notification; Consciousness). However, after comparing the items and dimensions of both versions, it can be observed that both versions address similar concepts. Thus, the new dimension Responsibility includes aspects of the dimensions Professional incompetence as the cause of errors and Disclosure responsibility; also, the new dimension Training unifies the dimensions Patient safety training received and Importance of patient safety in the curriculum. Furthermore, the previous dimension Patient involvement in reducing errors is observed in a transversal way in several new dimensions such as Organization and communication or Consciousness. In this way, the version of the APS questionnaire for nursing students obtained in this study simplifies the structure of the previous version, achieving a coherent and more parsimonious structure, with fewer items. These differences may be due to the idiosyncrasies of patient safety in each context [3]. In our case, it may also be because the questionnaires assess different professions, as was the case with the H-PEPSS questionnaire [14], initially validated with a sample of recent Canadian graduates in pharmacy, medicine and nursing but adapted and validated by Bressan et al. [16] for Italian nursing students, with relevant modifications in the structure and items of the questionnaire. In our case, an adequate factorial structure was confirmed, although some quality indicators of the fit were close to the minimum necessary value. Future studies with larger samples should improve this result.

At the time of this study, Mira et al. [40] validated a questionnaire to measure knowledge and attitudes towards patient safety in a sample of medical and nursing students from Spain and Latin America. The questionnaire is similar to the one in this study, composed of 21 items and 5 dimensions, and had good psychometric properties. Recently, Ortiz de Elguea et al. [41] adapted and validated the Hospital Survey on Patient Safety (HSOPS) questionnaire [42] using a sample of 654 Spanish nursing students; however, this questionnaire aims to measure the safety culture and not competencies about patient safety. The existence of these questionnaires provides an opportunity to progress in the search for a valid and reliable instrument that will allow evaluating the patient safety competencies of nursing students in Spain and Latin America.

Otherwise, student attitudes towards patient safety can be considered overall adequate; however, it should be mentioned that second-year students obtained overall scores significantly higher than those obtained by students in other academic years. Else, the score for the Training dimension also showed significant differences, with a progressive increase from the first year to the fourth year. This is notable because the desirable result would be that the scores progressively improve throughout the 4 academic years, indicating a progressive acquisition of competencies. Different authors [3, 43] recommend that competencies regarding patient safety be addressed throughout the different academic years and linked to experiences related to patient safety during clinical practicums [7]. In our studies, competencies in patient safety are addressed in the third year, and there is no progressive strategy available to acquire these competencies.

In Europe and Spain, at least 50 % of nursing student learning takes place in health centres under the supervision of clinical nurses [44]. In this way, students learn about patient safety in both contexts, integrating university content into the reality of care and observing the factors that influence patient safety [7, 18]. This may be why students who completed clinical practicums or had previous studies obtained significantly higher overall scores as well as higher scores in the Responsibility and Training dimensions and in some items related to the importance of teamwork and the organization of services. However, Lukewich et al. [15] found that students did not trust what they were learning in practicums on teamwork or the management of adverse effects. The influence of nurses who care for students, the learning environment or safety culture on the acquisition of these skills should be further explored [45, 46].

The results of this study should be considered with caution for several reasons. On the one hand, the sample size could be considered limited and. However, approaches to determining sample size in validation studies vary considerably [30]. In our case, a subject to item ratio approach was used, where the literature recommends between 5 and 10 subjects per item [30]. We obtained a ratio of 6.1 participants per item (29 items in the version adapted by the nominal group), so the sample size can be considered adequate for the purpose of the study.

On the other hand, conducting an EFA and a CFA with the same sample is not routine practice and may seem inappropriate. However, we specify in the analysis section that this procedure was based on previous studies since we started from a questionnaire already validated [31, 32]. This procedure in the same sample allows to test the validity of the implicit restrictions in the CFA that were not part of the EFA (for example, fixed cross-loads or uncorrelated errors) and to control the effects of the measurement error [32, 47]. However, it is necessary to confirm the structure of the APS questionnaire in new samples. For this, new CFA or structural equation models can be made.

Finally, the study was carried out in one institution with a non-randomized sample, so that the results on the attitudes of nursing students towards patient safety cannot be extrapolated. Something that draws attention to the sample is the overrepresentation of men among first-year students with respect to the general distribution of the sample and, in general, with respect to the nursing profession. We think that it may be a chance finding with no impact on the results. Moreover, a longitudinal study should be conducted with follow-up of a cohort during the 4 years of the nursing degree to study the evolution of attitudes towards patient safety. Despite these limitations, the results are considered of interest because there are few studies that address this issue in the Spanish context, and validated instruments in Spanish to assess the attitudes of nursing students towards patient safety are very scarce.

## Conclusions

The APS questionnaire adapted for Spanish nursing students is a valid and reliable instrument to measure attitudes towards patient safety; however, it is necessary to make advances in the study of its psychometric properties in broader samples and including other institutions.

The sample of nursing students studied has an adequate attitude towards patient safety. Clinical practicums influence the acquisition of competencies related to patient safety, and there is no progressive improvement of their attitudes throughout the 4 academic years. The development of a progressive strategy to ensure the acquisition of competencies on patient safety and the evaluation of specific educational interventions could help to improve the acquisition of competencies and their education on patient safety.

## Supplementary Information

**Additional file 1.**

**Additional file 2.**

## Data Availability

The datasets used and/or analysed during the current study are available from the corresponding author on reasonable request.
