# Supplementary material for: Validation of the attitudes to patient safety questionnaire for nursing students in the Spanish context
Source: BMC Nurs. 2021 Jun 19;20:101. doi: 10.1186/s12912-021-00634-y (PMC8214300; doi:10.1186/s12912-021-00634-y)
Supplement: Supplementary file 1 — Additional file 1. [file 12912_2021_634_MOESM1_ESM.doc]

**Additional file 1. Comparison of the three versions of the APS questionnaire**

| **Original version for medical students in English (Carruters et al, 2009)** | **Adapted version for medical students in Spanish (Lamponi et al, 2014)** | **Adapted version for nursing degree students in Spanish (nominal group)** |
| --- | --- | --- |
| 1. **Patient safety training received**  - My training is preparing me to understand the causes of medical errors - I have a good understanding of patient safety issues as a result of my undergraduate medical training. - My training is preparing me to prevent medical errors | 1. **Entrenamiento recibido en seguridad del paciente**  - Mi entrenamiento me está preparando para entender las causas del error en medicina. - Como resultado de mi formación de pregrado, tengo una buena comprensión de las cuestiones de Seguridad del Paciente. - Mi entrenamiento me está preparando para prevenir errores en medicina. | 1. **Entrenamiento recibido en seguridad del paciente**  - Mi entrenamiento me está preparando para entender las causas del error en la práctica enfermera. - Como resultado de mi formación de pregrado, tengo una buena comprensión de las cuestiones de Seguridad del Paciente. - Mi entrenamiento me está preparando para prevenir errores en la práctica enfermera. |
| 1. **Error reporting confidence**  - I would feel comfortable reporting any errors I had made, no matter how serious the outcome had been for the patient. - I would feel comfortable reporting any errors other people had made, no matter how serious the outcome had been for the patient. - I am confident I could talk openly to my supervisor about an error I had made if it had resulted in potential or actual harm to my patient. | 1. **Confianza en el reporte del error**  - Me sentiría cómodo reportando cualquier error que haya cometido, sin importar cuán serios hayan sido los resultados para el paciente. - Me sentiría cómodo reportando cualquier error que otras personas hayan cometido, sin importar cuan serios hayan sido los resultados para el paciente. - Estoy seguro de que podría hablar abiertamente con mi supervisor acerca de un error que yo haya cometido si el mismo resultó en daño potencial o real a mi paciente. | 1. **Confianza en la notificación del error**  - Me sentiría cómodo notificando cualquier error que haya cometido, sin importar la gravedad de los resultados para el paciente. - Me sentiría cómodo notificando cualquier error que otras personas hayan cometido, sin importar la gravedad de los resultados para el paciente. - Estoy seguro de que podría hablar abiertamente con mi supervisor acerca de un error que yo haya cometido si el mismo resultó en daño potencial o real a mi paciente. |
| 1. **Working hours as error cause**  - Shorter shifts for doctors will reduce medical errors. - By not taking regular breaks during shifts doctors are at an increased risk of making errors. - The number of hours doctors work increases the likelihood of making medical errors. | 1. **Horas de trabajo como causa de error***  - Turnos de trabajo más cortos para los médicos reducirán los errores en medicina. - Al no tomar descansos regulares durante los turnos los médicos aumentan el riesgo de cometer errores. - El número de horas que trabajan los médicos aumenta la probabilidad de cometer errores en medicina | 1. **Factores como causa de error**  - Me sentiría cómodo notificando cualquier error que haya cometido, sin importar la gravedad de los resultados para el paciente. - Me sentiría cómodo notificando cualquier error que otras personas hayan cometido, sin importar la gravedad de los resultados para el paciente - Estoy seguro de que podría hablar abiertamente con mi supervisor acerca de un error que yo haya cometido si el mismo resultó en daño potencial o real a mi paciente |
| 1. **Error inevitability**  - Even the most experienced and competent doctors make errors. - A true professional does not make mistakes or errors. - Human error is inevitable. | 1. **Inevitabilidad del error**  - Incluso los médicos más experimentados y competentes cometen errores. - Un verdadero profesional no comete errores. - El error humano es inevitable. | 1. **Inevitabilidad del error**  - Incluso los/as enfermeros/as más experimentados y competentes cometen errores. - Un verdadero profesional no comete errores. - El error humano es inevitable. |
| 1. **Professional incompetence as error cause**  - Most medical errors result from careless nurses. - If people paid more attention at work, medical errors would be avoided. - Most medical errors result from careless doctors. - Medical errors are a sign of incompetence. | 1. **Incompetencia profesional como causa de error**  - La mayoría de los errores en medicina derivan de enfermeras descuidadas. - Si las personas prestaran más atención en el trabajo, los errores en medicina serían evitados. - La mayoría de los errores en medicina derivan de médicos descuidados. - Los errores en medicina son un signo de incompetencia. | 1. **Incompetencia profesional como causa de error**  - La mayoría de los errores en la práctica asistencial derivan de médicos descuidados. - La mayoría de los errores en la práctica asistencial derivan de residentes descuidados. - La mayoría de los errores en la práctica asistencial derivan de enfermeros/as descuidado/as. - La mayoría de los errores en la práctica asistencial derivan de auxiliares de enfermería descuidados/as. - Los errores en enfermería son un signo de incompetencia. |
| 1. **Disclosure responsibility**  - It is not necessary to report errors which do not result in adverse outcomes for the patient. - Doctors have a responsibility to disclose errors to patients only if they result in patient harm. - All medical errors should be reported. | 1. **Responsabilidad en la comunicación**  - No es necesario reportar errores que no resulten en eventos adversos para el paciente. - Los médicos tienen la responsabilidad de comunicar el error al paciente solo si el mismo resultó en daño para ese paciente. - Todos los errores en medicina deberían ser reportados. | 1. **Responsabilidad en la comunicación al paciente**  - No es necesario informar al paciente de los errores que no resulten en efectos adversos para su persona. - El personal de enfermería tiene la responsabilidad de comunicar el error al paciente solo si éste causó daño. - Todos los errores en enfermería deberían ser informados. |
| 1. **Team functioning**  - Better multi-disciplinary teamwork will reduce medical errors. - Teaching teamwork skills will reduce medical errors. | 1. **Funcionamiento del equipo**  - Un mejor trabajo en equipo multidisciplinario reducirá los errores en medicina. - Enseñar habilidades de trabajo en equipo reducirá los errores en medicina. | 1. **Funcionamiento del equipo**  - Si las personas prestaran más atención en el trabajo, los errores en la práctica asistencial serían evitados. - Un mejor trabajo en equipo multidisciplinario reducirá los errores en la práctica asistencial. - Enseñar habilidades de trabajo en equipo reducirá los errores en la práctica asistencial. |
| 1. **Patient involvement in reducing error**  - Patients have an important role in preventing medical errors. - Encouraging patients to be more involved in their care can help to reduce the risk of medical errors occurring. | 1. **Compromiso del paciente en la reducción del error**  - Los pacientes tienen un rol importante en la prevención del error en medicina. - Estimular a los pacientes a estar más involucrados en su cuidado puede ayudar a reducir el riesgo de ocurrencia de los errores en medicina. | 1. **Compromiso del paciente en la reducción del error**  - Los pacientes tienen un rol importante en la prevención del error en la práctica asistencial. - Estimular la participación de los pacientes en su cuidado puede ayudar a reducir la posibilidad del error en la práctica enfermera. |
| 1. **Importance of patient safety in the curriculum**  - Teaching students about patient safety should be an important priority in medical students training. - Patient safety issues cannot be taught and can only be learned by clinical experience when qualified. - Learning about patient safety issues before I qualify will enable me to become a more effective doctor. | 1. **Importancia de la seguridad del paciente en el currículum**  - Enseñar a los estudiantes sobre seguridad del paciente debería ser una prioridad en el entrenamiento médico de pregrado. - Las cuestiones en seguridad del paciente no pueden ser enseñadas y solo pueden ser aprendidas mediante la experiencia clínica luego de la graduación. - Aprender sobre seguridad del paciente antes de graduarme me permitirá convertirme en un médico más efectivo. | 1. **Importancia de la seguridad del paciente en el currículum académico**  - Enseñar a los estudiantes sobre la seguridad del paciente debería ser una prioridad en la formación de pregrado. - Las cuestiones en seguridad del paciente no pueden ser enseñadas y solo pueden ser aprendidas mediante la experiencia clínica después de la graduación. - Aprender sobre seguridad del paciente antes de graduarme me permitirá convertirme en un enfermero/a más eficaz. |
