# Supplementary material for: Validation of the attitudes to patient safety questionnaire for nursing students in the Spanish context
Source: BMC Nurs. 2021 Jun 19;20:101. doi: 10.1186/s12912-021-00634-y (PMC8214300; doi:10.1186/s12912-021-00634-y)
Supplement: Supplementary file 2 — Additional file 2. [file 12912_2021_634_MOESM2_ESM.doc]

**Additional file 2: Attitudes to Patient Safety Questionnaire for Nursing Students**

| **Dimensions and items*** |
| --- |
| **1. Responsibility** |
| Most errors derive from careless physicians** |
| Most errors derive from careless residents** |
| Most errors derive from careless nurses** |
| Most errors derive from careless nursing assistants** |
| If more attention was paid at work, errors would be avoided |
| **2. Organization and communication** |
| Adequate communication with the patient decreases adverse effects |
| Adequate communication with the team decreases adverse effects |
| An adequate workload decreases adverse effects |
| Adequate organization decreases adverse effects |
| The participation of patients in their care decreases adverse effects |
| **3. Teamwork** |
| Better work in a multidisciplinary team will reduce errors |
| Teaching teamwork skills will reduce errors |
| Learning about safety will allow me to become a more effective nurse |
| **4. Training** |
| My training prepares me to understand the causes of errors |
| I have a good understanding of patient safety issues due to my training |
| My training is preparing me to prevent errors in practice |
| The most experienced and competent nurses make mistakes |
| **5. Notification** |
| I would feel comfortable reporting any mistake I made |
| I would feel comfortable reporting other people’s mistakes |
| **6.Conciousness** |
| A true professional does not make mistakes ** |
| It is not necessary to inform the patient of errors that do not result in adverse effects** |
| Errors should be communicated to the patient only if they caused harm** |

* Spanish version translated to English without transcultural adaptation

** Items written in the opposite direction and with the scores inverted
